# Comparative Investigation of Human Amniotic Epithelial Cells and Mesenchymal Stem Cells for Application in Bone Tissue Engineering

**DOI:** 10.1155/2015/565732

**Published:** 2015-03-05

**Authors:** Jiawen Si, Jiewen Dai, Jianjun Zhang, Sha Liu, Jing Gu, Jun Shi, Steve G. F. Shen, Lihe Guo

**Affiliations:** ^1^Department of Oral and Craniomaxillofacial Science, Ninth People's Hospital College of Stomatology, Shanghai Jiao Tong University School of Medicine, Shanghai Key Laboratory of Stomatology, Shanghai 200011, China; ^2^Institute of Biochemistry and Cell Biology, Shanghai Institutes for Biological Sciences, Chinese Academy of Sciences, Shanghai 200031, China

## Abstract

Emerging evidence suggests amniotic epithelial cells (AECs) as a promising source of progenitor cells in regenerative medicine and bone tissue engineering. However, investigations comparing the regenerative properties of AECs with other sources of stem cells are particularly needed before the feasibility of AECs in bone tissue engineering can be determined. This study aimed to compare human amniotic epithelial cells (hAECs), human bone marrow mesenchymal stem cells (hBMSCs), and human amniotic fluid derived mesenchymal stem cells (hAFMSCs) in terms of their morphology, proliferation, immunophenotype profile, and osteogenic capacity *in vitro* and *in vivo*. Not only greatly distinguished by cell morphology and proliferation, hAECs, hAFMSCs, and hBMSCs exhibited remarkably different signature regarding immunophenotypical profile. Microarray analysis revealed a different expression profile of genes involved in ossification along the three cell sources, highlighting the impact of different anatomical origin and molecular response to osteogenic induction on the final tissue-forming potential. Furthermore, our data indicated a potential role of FOXC2 in early osteogenic commitment.

## 1. Introduction

With the progress of regenerative medicine, especially in the field of stem cells and biomaterials, stem cell-based bone tissue engineering has been recognized as a promising strategy for reconstruction of bone defects resulting from trauma, congenital malformations, and surgical resections [1–4]. Mesenchymal stem cells, more recently described as mesenchymal stromal cells (MSCs), have been successfully isolated from various regions of the body and have been suggested as a promising stem cell source for bone tissue engineering evidenced by extensive* in vitro* and* in vivo* studies [4–9]. However, the drawback of MSCs in invasive cell collection, aging, and limited cell quantity may restrict the utility of MSCs in further clinical practice, prompting increasing interests in alternative stem cells sources for bone tissue engineering [2, 5, 9, 10].

Emerging evidences have suggested that human placentas which are normally discarded after delivery constituted valuable sources of maternal and fetal cells that exhibit superior plasticity [7, 8, 11, 12]. Particular attention has been directed to human amniotic epithelial cells (hAECs) as a source of progenitor cells of fetal origin with no ethical issue involvement. Previous and extensive studies have shown that amniotic epithelial cells from different species such as rat, sheep, and human possess combined qualities of both embryonic and adult stem cells and retain a remarkable plasticity [13–17]. HAECs have been shown to possess trilineage differentiation ability* in vitro* and express markers of both mesenchymal and embryonic stem cells (ESCs) [11, 14, 17, 18]. In contrast to ESCs, hAECs have been shown to display a stable nontumorigenic phenotype, evidenced by several long-term* in vivo* transplantation experiments [11, 13, 14]. Furthermore, the fetal origin may provide hAECs with not only the fetus-maternal immunotolerance but also an immunomodulatory property, thus supporting the application safety of hAECs in allotransplantation [19–21]. All these attractive characteristics make hAECs a promising and noncontroversial source of progenitor cells for extensive use in cell transplantation and regenerative medicine.

Very recently, the* in vitro* and* in vivo* osteogenic ability of amniotic epithelial cells was demonstrated in various studies indicating that amniotic epithelial cells may be an appropriate source of progenitor cells for bone tissue engineering [12, 15, 18, 22]. However, further systemic investigations comparing the regenerative properties of hAECs with other sources of stem cells are particularly needed before the feasibility of hAECs in bone tissue engineering can be determined [18, 22]. In light of the findings of recent research progress, we have isolated hAECs, human bone marrow mesenchymal stem cells (hBMSCs), and human amniotic fluid derived mesenchymal stem cells (hAFMSCs), respectively, and compared these cells on the basis of their morphology, proliferation, immunophenotype profile, and osteogenic differentiation potential* in vitro* and* in vivo*. To the best of our knowledge, the data reported here for the first time documented the similarities and differences of hAECs, hBMSCs, and hAFMSCs, which may provide important application guidance of cells originating from these three distinct sources in bone tissue engineering.

## 2. Materials and Methods

### 2.1. Isolation and Culture of Cells

Human amnion membranes were obtained from healthy mothers undergoing cesarean sections; human amniotic fluid was obtained by ultrasound-guided amniocentesis performed on pregnant women for routine prenatal diagnosis purposes at gestational ages ranging from 18 to 22 weeks; human bone marrow was obtained from women patients with alveolar cleft undergoing autogenous bone grafting. All subjects agreed with the written informed consent and were negative for HIV-I, hepatitis B, and hepatitis C. The appropriate use of human tissue and cells in this study was approved by the institutional patients and ethics committee. The hAECs, hBMSCs, and hAFMSCs were prepared and isolated as we previously described [23–25]. After isolation, the hAECs, hBMSCs, and hAFMSCs were cultured in the expansion culture medium (EXP-CM) and expanded to passage 2 before further study. The EXP-CM for hAECs was prepared with DMEM/F12 medium (Invitrogen, China) supplemented with 2 mM L-glutamine (Invitrogen, China), 10 ng/mL rhEGF (Invitrogen, China), 20% fetal bovine serum (Gibco, China), and 1% penicillin-streptomycin (Invitrogen, China), while the EXP-CM for hBMSCs and hAFMSCs differs only in the added growth factor with additional 10 ng/mL rhFGF (Invitrogen, China).

### 2.2. *In Vitro* Comparison of Cells Morphology and Proliferation

HAECs, hBMSCs, and hAFMSCs were cultured on both 24-well plates and previously described microroughened titanium discs in EXP-CM [25]. All samples were washed with PBS and fixed in 2.5% w/v glutaraldehyde (Sigma-Aldrich, USA) overnight. Morphology of the adherent cells on plates was photographed using a light microscope (Axio Scope A1, Zeiss, Germany) provided with a digital camera (SPOT Flex, SPOT, USA). After a graded dehydration and gold sputter-coating, the morphology of the adherent cells on the titanium discs was observed by scanning electron microscopy (SEM).

HAECs, hBMSCs, and hAFMSCs were seeded at low density (1 × 10^3^ cells/well) into a 96-well plate and cultured for 4 hours (hrs), 2, 4, 6, 8, 10, and 12 days, respectively. At each predetermined time point, cell proliferation was compared using a Cell Counting Kit-8 (CCK-8, Dojindo, Japan) according to the manufacturer's recommendation. Triplicate samples were tested in each group at each incubation time.

### 2.3. Flow Cytometric Analysis

Semiconfluent cultures of hAECs, hBMSCs, and hAFMSCs were harvested with trypsin/EDTA (Invitrogen, China) and washed with PBS containing 0.5% bovine serum albumin (BSA). For examination of basic surface markers expression, 1 × 10^6^ hAECs, hBMSCs, and hAFMSCs were incubated with the following phycoerythrin (PE) or fluorescein isothiocyanate (FITC) conjugated anti-human primary antibodies (all purchased from Miltenyi, Germany) for 30 min at 4°C according to the manufacturer's recommendation: PE-CD44, FITC-CD45, PE-CD90, FITC-CD34, PE-CD105, FITC-stage-specific embryonic antigen (SSEA) 4, and FITC-SSEA3. For examination of immunologic surface markers expression, 1 × 10^6^ hAECs, hBMSCs, and hAFMSCs cultured with or without 10 ng/mL interferon-*γ* (IFN-*γ*) for 5 days were incubated with the following PE or FITC conjugated anti-human primary antibodies (all purchased from Biolegend, USA) in the dark for 30 min at 4°C, respectively: FITC-human leukocyte antigen- (HLA-) ABC, PE-HLA-DR, PE-HLA-G, PE-HLA-E, PE-programmed death ligand 1 (PD-L1), PE-PD-L2, FITC-Toll-like receptor 5 (TLR5), PE-TLR6, FITC-Fas, and PE-Fas ligand (FasL). Experiments were performed in triplicate for each group. Nonspecific fluorescence was gated by using respective isotype-matched monoclonal-antibody controls. Flow cytometry data were analyzed using a Beckman Coulter EPICS XL cytometer (Beckman Coulter, USA) equipped with a FlowCentre workstation (Beckman Coulter, USA).

### 2.4. *In Vitro* Differentiation and Biochemical Assay

#### 2.4.1. *In Vitro* Osteogenic Differentiation

HAECs, hBMSCs, and hAFMSCs at 70%–80% confluence in test wells were cultured in a classical osteogenic induction medium (*α*-MEM (Invitrogen, China) supplemented with 10% FBS (Gibico, China), 0.1 mM ascorbic acid (Sigma-Aldrich, USA), 10 mM *β*-glycerophosphate (Sigma-Aldrich, USA), and 10^−5^ mM dexamethasone (Sigma-Aldrich, USA)). The osteogenic induction medium was changed every 3 days and the experiments were terminated at day 21. Cells cultured in EXP-CM were set as control. Experiments were performed in triplicate for each group.

#### 2.4.2. Alkaline Phosphatase (ALP) Activity Assessment

After osteogenic differentiation for 5 and 10 days as described above, cells in each group were fixed with 4% paraformaldehyde solution for 10 min at room temperature (RT) and washed with PBS twice. An ALP staining kit (Beyotime, China) was used for ALP staining according to the manufacturer's instructions. Then all samples were rinsed with PBS and photographed.

The ALP activity of hAECs, hBMSCs, and hAFMSCs in the osteogenic medium at days 5 and 10 was measured using a p-nitrophenyl phosphate (pNPP) (Sigma-Aldrich, USA) method [26]. Briefly, the cells in each well were washed with PBS and lysed by incubation of 500 *μ*L of 0.1% Triton X-100 (Sigma-Aldrich, USA) in 10 mM Tris-HCl (pH 7.4) for 2 h at 4°C. 50 *μ*L lysate of each sample was mixed with 50 *μ*L pNPP (1 mg/mL) and incubated at 37°C for 15 min in a 96-well culture plate. The ALP activity was quantified by measuring the light absorbance at 405 nm using a plate reader. The ALP activity was normalized according to the level of total protein content at each time point using a BCA kit (Thermo Scientific Pierce Protein Biology Products, USA). Experiments were performed in triplicate for each group.

#### 2.4.3. Alizarin Red S (ARS) Staining

Extracellular calcium deposition of cells in each group at day 21 was determined using ARS staining. The cell layers were fixed with 4% paraformaldehyde solution for 20 min at RT and washed with pure water twice. All samples were incubated in 40 mM ARS (pH 4.2; Sigma-Aldrich, USA) solution for 15 min, washed with pure water, and photographed.

Then a semiquantitative Alizarin red stain (ARS) assay was used to further compare the extracellular calcium deposition in each group [26]. The Alizarin red in each sample was destained in 10 mM sodium phosphate containing 10% cetylpyridinium chloride (pH 7.0; Sigma-Aldrich, USA), for 15 min at RT. The reaction products were transferred to a 96-well culture plate and determined by measuring the light absorbance at 562 nm using a plate reader. Experiments were performed in triplicate for each group.

### 2.5. Microarray Analysis

Global gene expression profiles of hAECs, hBMSCs, and hAFMSCs were evaluated before and after 7-day osteogenic induction* in vitro*. Samples were subjected to gene expression analysis using the Affymetrix human HTA2.0 microarray (Affymetrix, USA) according to the manufacturer's recommendations (for details, see Supplement Material I in the Supplementary Material available online at http://dx.doi.org/10.1155/2015/565732).

### 2.6. Real-Time PCR and Western Blot

HAECs, hBMSCs, and hAFMSCs cultured in the osteogenic medium and EXP-CM were harvested at 0, 7, 10, and 14 days after osteogenic induction. Total RNA was extracted with RNAiso Plus reagent (Takara, Japan) and equivalent amount of each RNA sample was reverse-transcribed into cDNA using a PrimeScript RT-PCR Kit (Takara, Japan) according to the product sheet provided by the manufacturer. The expression levels of* Runx2, Osterix (OSX), Collagen I (COLI), ALP, Osteopontin (OPN), BMP2, BMP4, BMP6, FOXC1, FOXC2,* and* GAPDH* at each time point were determined quantitatively on a real-time PCR machine (ABI 7300, USA) using a SYBR Premix Ex Taq kit (TaKaRa, Japan), with GAPDH as the housekeeping gene for normalization. Details of primers are listed in Supplement Material II. Data were analyzed using the comparative CT method and expressed as the fold change.

To verify the upregulation of FOXC2 during osteogenesis, HAECs, hBMSCs, and hAFMSCs were treated with 0, 25, 50, 75, 100, and 200 ng/mL rhBMP2 (PeproTech, USA), a wildly accepted bone formation inducing cytokine, for 3 days and subjected to real-time PCR for detection of FOXC2 expression. Then, all three cells were treated with 100 ng/mL rhBMP2 (PeproTech, USA) and harvested at 0, 1, 3, 7, and 10 days. Samples were subjected to both real-time PCR and western blot for detection of FOXC2 expression, respectively. For western blot, cells were lysed with a commercial sodium dodecyl sulfate cell lysis buffer (Beyotime, China) supplemented with phosphatase inhibitors (I and II) and protease inhibitors (Sigma, USA) at 0, 1, 3, 7, and 10 days after BMP2 treatment. Cells treated with osteogenic induction medium were set as the control. Then all protein extracts were separated by 10% sodium dodecyl sulfate polyacrylamide gel electrophoresis (SDS-PAGE; Bio-Rad, USA) and subjected to western blot with primary rabbit polyclonal antibody against FOXC2 (1 : 1000 Abcam, UK). Images of western blot were taken with an Odyssey infrared imaging system (LI-COR bioscience, USA).

### 2.7. Cytoimmunofluorescence

HAECs, hBMSCs, and hAFMSCs cultured in the osteogenic medium and EXP-CM for 10 days were fixed with 4% paraformaldehyde solution and permeabilized with 0.1% Triton X-100. Nonspecific binding sites were blocked with 5% BSA. After coincubation with primary rabbit polyclonal anti-human OPN antibodies (1 : 100, Proteintech Group, USA) and primary mouse monoclonal anti-human Runx2 antibodies (1 : 100, ProSci Incorporated, USA) over night, the samples were washed and coincubated with Cy3-labeled goat anti-rabbit IgG (1 : 1000, Beyotime, China) and FITC-labeled goat anti-mouse IgG (1 : 1000, Beyotime, China). At the end of the incubation, the samples were counterstained with DAPI (1 : 5000, Beyotime, China). The Cy3, FITC, and DAPI images were taken separately using a fluorescence microscope (DP72; Olympus, Japan) equipped with a digital image capture system (Olympus).

### 2.8. Ectopic Osteogenesis in Nude Mice 

#### 2.8.1. Cells/Scaffold Constructs Assembly and Surgical Procedure

The animal study protocol was approved by the Institutional Animal Research Ethics Committee. Commercial available beta-tricalcium phosphate (*β*-TCP) scaffolds (diameter, 5 mm; height, 10 mm) were purchased from Shanghai Ceramic Institute of Chinese Academy of Sciences. The hAECs, hBMSCs, and hAFMSCs labeled with lenti-GFP were cultured in osteogenic medium for 7 days and seeded on the scaffolds as previously described [1, 6]. Four dorsal subcutaneous pockets were formed as previously described on each anesthetized female nude mouse for insertion of the following 4 groups of constructs: a *β*-TCP scaffold construct, a *β*-TCP/hAECs construct, a *β*-TCP/BMSCs construct, and a *β*-TCP/hAFMSCs construct [6, 27]. Experiments were performed in triplicate for each group.

#### 2.8.2. Histological Analysis

At 4 weeks after surgical operation (postop), animals were humanely killed under deep anesthesia. Samples were extracted and fixed in 4% paraformaldehyde. After decalcification, the specimens were embedded in paraffin and sectioned into 4 *μ*m thick sections. For morphological study, sections were stained with hematoxylin and eosin (HE) with an autostainer (ST5010, Leica, Germany). For immunohistochemistry analysis, sections were subjected to immunostaining with mouse monoclonal anti-GFP (1 : 100, Santa Cruz, USA), rabbit polyclonal anti-rat osteocalcin (OCN) (1 : 100, Santa Cruz, USA), and rabbit polyclonal anti-human OPN antibodies (1 : 100, Proteintech Group, USA). Then all samples were photographed.

### 2.9. Statistical Analysis

All measurements were collected and expressed as mean ± standard deviation (SD). Data for these measurements were analyzed using two-way ANOVA and Student's *t*-test. A *P* value < 0.05 was considered statistically significant. SPSS 16.0 software and Graphpad prism 5.0 software were utilized to analyze and demonstrate the statistical significance of the assays. The significance between groups was marked on the graphs.

## 3. Results

### 3.1. hAECs Show a Different Phenotype Compared to hBMSCs and hAFMSCs

**Figure 1:**
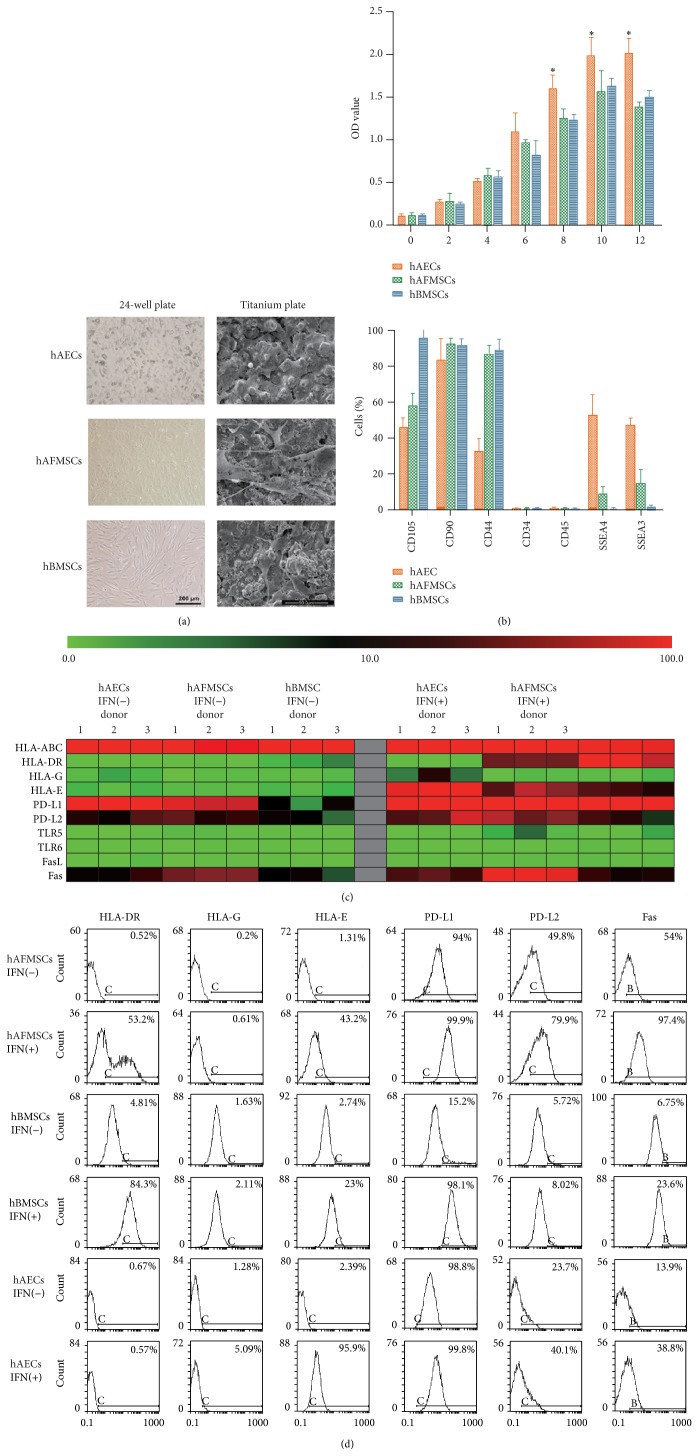
hAECs show a different phenotype compared to hBMSCs and hAFMSCs. (a) hAECs exhibited an epithelial-like morphology under the light microscope and scanning electron microscope, while both hAFMSCs and hBMSCs showed a spindle-shaped fibroblast morphology. (b) CCK-8 results at days 0, 2, 4, 6, 8, 10, and 12 after cell seeding revealed a significant higher proliferation activity of hAECs at day 8, 10, and 12 compared to the other two cells (∗: *P* < 0.05). Flow cytometry analysis for the basic surface makers demonstrated that all cell sources were positive for the MSC markers CD44, CD90, CD105, and lacked the expression of hematopoietic makers CD45, CD34. Moreover, hAECs expressed a higher level of CD326 and SSEA4, while hBMSCs barely expressed these markers. (c) After culturing with or without 10 ng/mL IFN-*γ* for 5 days, expression patterns of immunologic markers in all cell types are presented as heat maps of the percentage of cells in the total population expressing the marker (see color legend). (d) Flow cytometry results for HLA-DR, HLA-G, HLA-E, PD-L1, PD-L2, and Fas were selectively shown. Nonspecific fluorescence was gated by using respective isotype-matched monoclonal-antibody controls.

The primary adherent cells of hAECs, hBMSCs, and hAFMSCs normally reached confluence after 10–14 days in culture. After passaging, hAECs were successfully generated with homogeneous cobblestone-like morphology while hBMSCs and hAFMSCs showed spindle-shaped fibroblast morphology (Figure 1(a)). When cultured on the microroughened titanium coatings, both hBMSCs and hAFMSCs showed a well spread spindle-like morphology with presence of classical pseudopodia, while hAECs showed much shorter pseudopodia and closer cell-cell contacts (Figure 1(a)).

The proliferation of hAECs, hBMSCs, and hAFMSCs was determined using a CCK-8 assay. All three cell types proliferate with time, whereas cell proliferation of hAECs at days 8, 10, and 12 was significantly higher compared to the other two cells (*P* < 0.05) (Figure 1(b)).

Flow cytometry analysis for basic surface makers demonstrated that all cell sources were positive for the MSC markers CD44, CD90, and CD105 and lacked the expression of hematopoietic makers CD45, CD34 (Figure 1(b) and Table 1). Interestingly, 52.47% ± 11.82 hAECs and 8.10% ± 4.84 hAFMSCs expressed SSEA4, while 46.87% ± 4.30 hAECs and 14.73% ± 8.00 hAFMSCs expressed SSEA3, both of which have been classic embryonic stem cells specific markers, while hBMSCs barely expressed these markers (Table 1).

Further analysis for immunologic surface markers was summarized in Table 1 and selectively presented in Figures 1(d) as both heat maps and flow cytometry histograms of the percentage of cells expressing the marker. More than 95% of all cell sources expressed HLA-ABC while less than 5% of all cell sources expressed HLA-DR, HLA-G, HLA-E, TLR5, TLR6, and FasL. Both hAECs and hAFMSCs expressed a significant higher level of PD-L1 and PD-L2 than hBMSCs while hBMSCs expressed the lowest level of Fas. After IFN-*γ* treatment, the expressions of HLA-E, PD-L1, PD-L2, and Fas in all cell sources were significantly upregulated while the expressions of HLA-ABC, TLR5, TLR6, and FasL were barely affected. Interestingly, IFN-*γ* treatment significantly upregulated the HLA-DR in hBMSCs and hAFMSCs, while it failed to affect the expression of HLA-DR in hAECs. In contrast, HLA-G, the nonclassical HLA class I molecule that plays a role in maternofetal tolerance, was significantly upregulated by IFN-*γ* treatment only in hAECs.

### 3.2. hAECs Show a Confirmed Though Relative Lower Osteoblastic Capacity* In Vitro*

**Figure 2:**
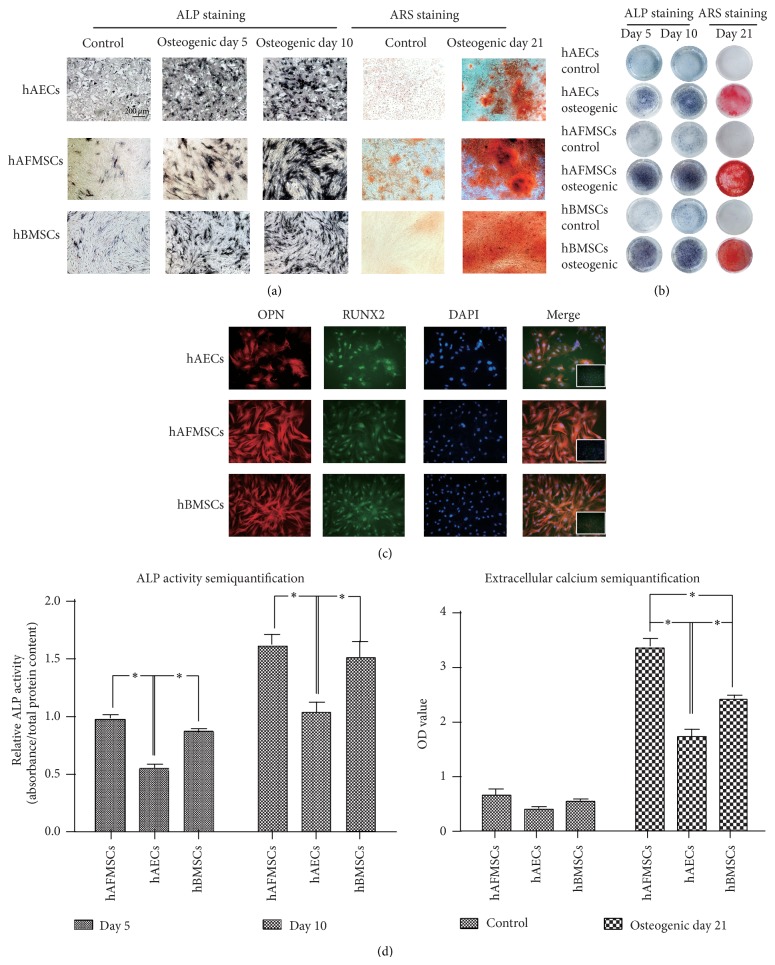
hAECs show a confirmed though relative lower osteoblastic capacity* in vitro*. (a, b) Progressively increased cellular ALP and ARS staining after osteogenic induction were observed in all cell types. (c) Immunofluorescence labeling of Runx2 (FITC, green), OPN (Cy3, red), and Nucleus (DAPI, blue) in hAECs, hBMSCs, and hAFMSCs following the 10-day osteogenic induction exhibited a more intense fluorescence of OPN and clear nuclei-localization of RUNX2 (small images at the corner of the merged images indicated the merged images of control group). (d) Further semiquantification of ALP activity and extracellular mineralization showed the lowest cellular ALP activity and mineral producing efficiency in hAECs group while hAFMSCs exhibited the highest level of extracellular mineralization (c; ∗: *P* < 0.05).

ALP and ARS staining confirmed the progressively increased cellular ALP activity and extracellular mineralization after osteogenic induction in all cell types (Figures 2(a) and 2(b)). In addition, the cytoimmunofluorescence of Runx2 and OPN further confirmed the osteoblastic phenotype of all three cell sources, characterized by intense fluorescence of OPN and nuclei-localization of* Runx2* (Figure 2(c)). Further semiquantification of the ALP activity and extracellular mineralization demonstrated that both ALP activity and extracellular mineralization in all cell sources increased with elapsed time after osteogenic induction. However, the ALP activity and extracellular mineralization of hAECs at each determined time point showed a significant lower level (*P* < 0.05) than those in hBMSCs and hAFMSCs (Figure 2(d)).

### 3.3. hAECs, hBMSCs, and hAFMSCs Show a Different Molecular Response to Osteogenic Induction

**Figure 3:**
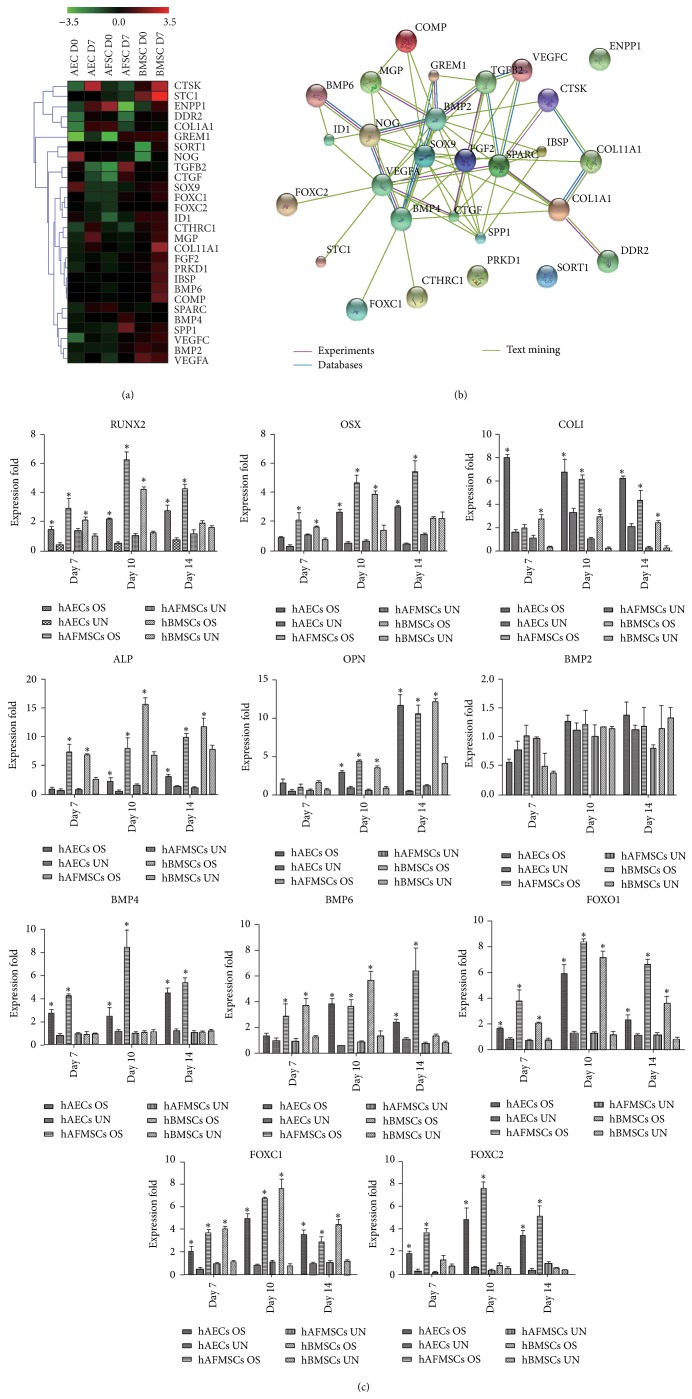
hAECs, hBMSCs, and hAFMSCs show a different molecular response to osteogenic induction* in vitro*. (a) Hierarchical cluster analysis of the differentially expressed genes involved in ossification. (b) Protein-protein interaction network analysis of the genes upregulated in hAECs, hBMSCs, and hAFMSCs after the 7-day osteogenic induction. (c) RUNX2, OSX, COLI, ALP, OPN, BMP6, FOXO1, and FOXC1 in osteogenic groups were gradually upregulated with time compared to those in the control group, while BMP4 and FOXC2 were only significantly upregulated in hAECs and hAFMSCs but not hBMSCs (∗: *P* < 0.05).

Microarray analysis of genes differentially expressed in hAECs, hBMSCs, and hAFMSCs before and after osteogenic induction revealed 65 genes involved in ossification (for details, please access the GEO database, http://www.ncbi.nlm.nih.gov/geo/query/acc.cgi?acc=GSE57265). Hierarchical cluster analysis of the microarray results demonstrated that genes involved in ossification were differentially expressed in hAECs, hBMSCs, and hAFMSCs indicating a relative different molecular response to osteogenic induction (Figure 3(a)). In fact, many differentially expressed genes showed higher expression in hBMSCs than the other two cell sources even before osteogenic induction, meeting the current definitions of the osteolineage restricted bone-marrow-derived MSCs [28]. The protein-protein interaction network analysis of genes upregulated in hAECs, hBMSCs, and hAFMSCs after osteogenic induction revealed 13 hub genes:* GREM1, TGF*β*2, NOG, BMP2, BMP4, SOX9, FGF2, SPARC, VEGFA, COL1A1, COL11A1, CTGF,* and* SPP1* (Figure 3(b)).

### 3.4. Expression of FOXC2 Is Upregulated during Osteogenic Differentiation

To confirm the osteoblastic phenotype and microarray results in all three cell sources, the expression of selective osteogenic specific genes was verified by real-time PCR. In detail, the expression of* Runx2, OSX, COLI, ALP, OPN, BMP6, FOXO1,* and* FOXC1* in osteogenic groups was gradually upregulated with time compared to those in the control groups (*P* < 0.05), while no differences of BMP2 expression were observed among the groups (Figure 3(c)). Interestingly, the expression of* BMP4* and* FOXC2* was significantly upregulated in hAECs and hAFMSCs but not hBMSCs following the osteogenic induction, indicating a nonclassical role of the two genes in osteogenesis.

**Figure 4:**
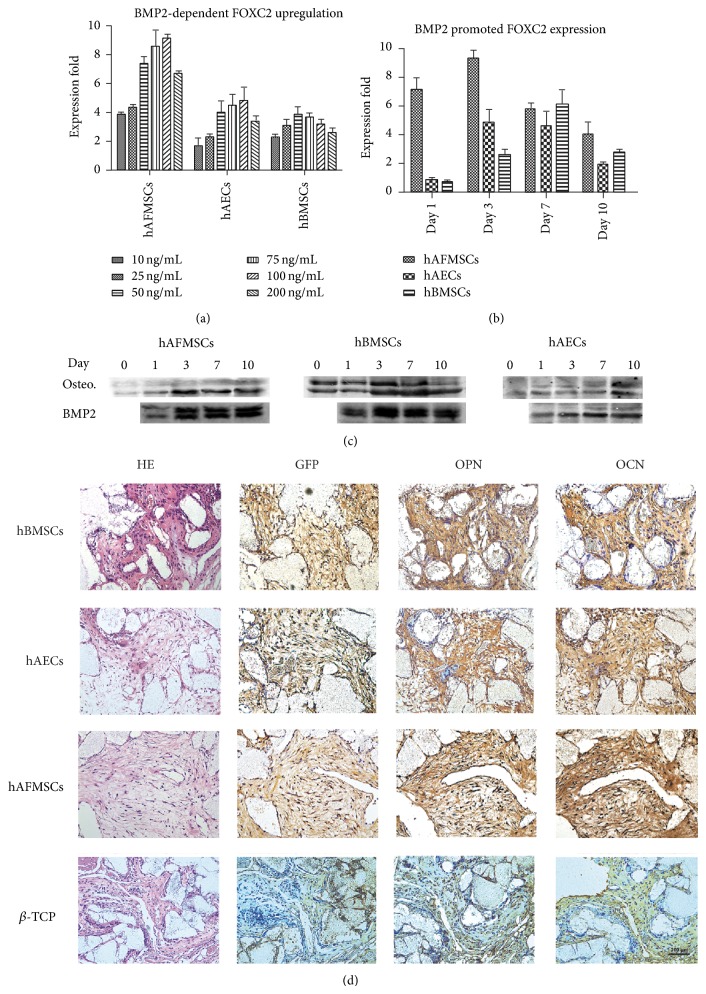
Verification of the upregulation of FOXC2 and ectopic osteogenesis of hAECs, hBMSCs, and hAFMSCs. (a) Expression of FOXC2 in all three cell types was significantly increased in a BMP2-dependent manner. (b, c) Both real-time PCR and western blot revealed that BMP2 significantly promoted the expression of FOXC2 in all the three cell sources. The western blot study also demonstrated that undifferentiated hBMSCs exhibited a higher expression level of FOXC2 than the other two cell sources. (d) Ectopic osteogenesis of hAECs, hBMSCs, and hAFMSCs in nude mice. *β*-TCP scaffolds carrying hAECs, hAFMSCs, and hBMSCs or alone were implanted subcutaneously for 4 weeks. HE staining showed no well-mineralized islands in either experimental groups or control group. Immunohistochemical staining showed that all cell types were viable as indicated by the positive expression of GFP. Moreover, OPN and OCN were evident in the experimental groups but not in the control group.

To further confirm the upregulation of FOXC2 during osteogenic differentiation, the expression of FOXC2 in hAECs, hBMSCs, and hAFMSCs following BMP2 treatment was examined through real-time PCR study and western blot. In detail, the expression of FOXC2 in all three cell sources was significantly increased in a BMP2-dependent manner (Figure 4(a)). BMP2 significantly promoted the early expression of FOXC2 in all three cell sources (Figures 4(b) and 4(c)). The western blot study also demonstrated that undifferentiated hBMSCs exhibited a higher expression level of FOXC2 than the other two cell sources, indicating a potential role of FOXC2 in early formation of the osteolineage related mesenchymal tissues (Figure 4(c)).

### 3.5. hBMSC, hAEC, and hAFSC Promoted the Ectopic Osteogenesis* In Vivo*

The samples were extracted at 1 month after implantation. Cell tracing results with GFP demonstrated that a portion of all three cell sources was still viable. Although the HE staining did not show the sign of well-mineralized islands formed both in experimental groups and *β*-TCP control group, the immunohistochemical staining showed that OPN and OCN were expressed at a higher level in all experimental groups compared with the control group, indicating the osteogenic potential of all three cell sources. In addition, our immunohistochemical assay showed that the expression of OPN and OCN was stronger in hBMSC group, while the OPN and OCN staining in hAEC group and hAFSC group were much more limited (Figure 4(d)).

## 4. Discussion

Human amniotic epithelial cells have been drawing increasing interest as a source of progenitor cells for regenerative medicine based on their phenotypic plasticity, immunomodulatory properties, and ready availability with no ethical issue involvement [14, 15, 18, 22, 29]. In addressing the current need for comparing the regenerative properties of hAECs for bone engineering with other sources of stem cells, we compared hAECs with hAFMSCs and hBMSCs in terms of cell morphology, proliferation, immunophenotypical profile, and osteogenic differentiation capacity. Our results shown here strongly validated the* in vitro* and* in vivo* osteogenic capacity of all three cell sources and for the first time demonstrated that hAECs possessed a better immunomodulatory but less osteogenesis capacity than hAFMSCs and hBMSCs.

HAECs, hAFMSCs and hBMSCs exhibited remarkably different signature regarding cell morphology, proliferation and immunophenotypical profile. In terms of the basic panel, hAECs expressed mesenchymal stem cells markers such as CD44, CD90, and CD105, as well as a remarkably higher level of SSEA4 and SSEA3 comparing to hAFMSCs and hBMSCs, which indicated a potentially more multipotent character in hAECs [14, 29]. As stem cells, although manufactured under favorable conditions, should be subsequently subjected to a damaged/diseased environment, immune response of the cells to certain proinflammatory cytokines such as IFN-*γ* could have a significant influence on the final effectiveness of regenerative therapy [10]. In terms of the immunologic panel, all three cell sources barely exhibited HLA-DR, HLA-G, HLA-E, TLR5, TLR6, and FasL, while IFN-*γ* treatment significantly upregulated the expression of HLA-DR and HLA-E in hAFMSCs and hBMSCs and the expression of HLA-G and HLA-E in hAECs. Also, our results indicated that hAECs and hAFMSCs exhibited a significant higher level of PD-L1 and PD-L2 than hBMSCs, while IFN-*γ* treatment significantly upregulated the expression of both markers in hBMSCs. However, the expression level of HLA-E and PD-L2 in hBMSCs was still remarkably lower than in hAECs and hAFMSCs even after IFN-*γ* treatment. Interestingly, HLA-G, HLA-E, PD-L1, and PD-L2 have been found to play key roles in maternal-fetus immunotolerance in various studies [20, 30, 31]. In fact, it was found that both PD-L1 and PD-L2 may play key roles in both T-cell- and B-cell-mediated immunotolerance, as inhibiting antigen-stimulated cell activation and proinflammatory cytokines production [31]. In addition, the tissue-restricted, nonclassical HLA class I antigen HLA-G has also been shown to possess substantial immunomodulatory functions in maternal tolerance of the fetus by mediating protection from the deleterious effects of natural killer cells, cytotoxic T-lymphocytes, macrophages, and mononuclear cells [31, 32]. According to the recent work of Lim et al., term hAECs exerted significantly more protective effects than preterm hAEC following acute lung injury partly through higher levels of HLA-G [32]. Taken together, these marker diversities between three cell sources pointed to a superior immunomodulatory property in hAECs, even without considering the long-term proved immunoprivileged and anti-inflammatory properties of hAECs* in vitro* and* in vivo* [13, 16, 19–21].

When analyzing the* in vitro* osteogenic differentiation potential of hAECs, hAFMSCs, and hBMSCs, we found that hAECs displayed a confimed osteoblastic differentiation capacity, while hAFMSCs and hBMSCs showed a higher osteoblastic phenotype. The remarkably different plasticity of all three cell sources under osteogenic induction condition, in agreement with previous reports in the literature, highlights the impact of the ontological and anatomical origin on the final tissue-forming potential [7, 11, 12, 14, 15, 18, 22, 33–35]. One plausible explanation for our result is that the different dynamic microenvironments or the stem cell niches may play an important role in modulating the behavior of each cell source [36, 37]. The term “niche” was firstly used by Schofield in 1978 to explain the variation in the self-renewal ability of apparently pure populations haemopoietic stem cells [38]. After decades of study, this concept has been extended to involve direct interactions between stem cells and neighboring cells, secreted factors, inflammation, extracellular matrix, physical parameters, and environmental signals [39]. In fact, our results, showing a more mature and wide-spread osteoblastic differentiation in hBMSCs than in hAECs and hAFMSCs, met the current definitions of the osteolineage restricted bone-marrow-derived MSCs [28]. Alternatively, it may be due to the different responsiveness of perinatal stem cells towards the osteogenic induction indicated by the differences of global genes profile between three cell sources. Of special interest is the upregulated expression of FOXC2 in hAECs and hAFMSCs. Undifferentiated hBMSCs exhibited a remarkably higher expression of FOXC2 than the other two cell sources, while osteogenic induction barely affected its expression in hBMSCs. Consistent with previous reports [40, 41], our results also confirmed that the upregulation of FOXC2 can be promoted by BMP2 in all three cell sources. In fact, several studies have described the essential roles of FOXC2 in skeletal development and osteogenesis, including the capability to enhance osteogenic differentiation in various cells and indispensable roles in neurocranium and vertebrae development [42–45]. Interestingly, FOXC2 has also been reported to inhibit adipogenic differentiation and was associated with bone mineral density in community-dwelling Japanese individuals [46–48]. As the reciprocal relationship between osteogenesis and adipogenesis during mesodermal differentiation has been most clearly elucidated, our observation together with previous studies indicates a potential key role of FOXC2 in early osteoblastic phenotype commitment and bone metabolism.

To further investigate the osteogenic capacity of all three cell sources* in vivo*, GFP labeled hAECs, hAFMSCs, and hBMSCs were seeded onto *β*-TCP scaffolds and implanted subcutaneously into the nude mice. The immunohistochemical analysis showed that the expression of OPN and OCN was remarkably upregulated in all three experiment groups (hAECs, hAFMSCs, and hBMSCs combined with *β*-TCP, resp.) comparing to the control group, which indicated the* in vivo* osteogenic capacity of all three cell sources [6, 27]. However, as multiple factors in the subcutaneous region could influence the fate of* in vivo* lineage commitment of progenitor cells, we did not further compare the* in vivo* osteogenic capacity using this ectopic osteogenesis model [27, 49, 50]. In fact, the osteogenic differentiation of hAECs, hAFMSCs, and hBMSCs has been investigated using various animal models in previous studies, which strongly confirmed the bone regenerative properties of all three cell sources* in vivo* [6, 7, 15, 17, 18, 33–35]. These results as well as ours suggest that further studies compare the regenerative potential of hAECs with other stem cells in a larger animal model.

Given the growing evidence showing that the transcriptome of even seemingly homogenous stem cell cultures may be extremely variable, we employed unfractioned hAECs, hAFSCs, and hBMSCs in this study, due to the lack of specific cell markers for the cell-sorting as well as the difficulties in maintaining and further application of the pure stem cell subpopulation according to the recent work of Hough et al. [51]. Overall, this is the first study reporting the comparison of hAECs, hAFMSCs, and hBMSCs regarding their immunophenotype profile and osteogenic capacity. The relative different osteoblastic capacity of all three cell sources highlights the impact of different anatomical origin and molecular response to osteogenic induction on the final tissue-forming potential. Furthermore, our data indicated a potential role of FOXC2 in early osteogenic commitment.

## Supplementary Material

Microarray analysis of genes differentially expressed in hAECs, hBMSCs and hAFMSCs before and after osteogenic induction revealed 65 genes involved in ossification.

## Figures and Tables

**Table 1:** Comparison of cells immunophenotype profiles.

	Basic surface markers panel				Immunologic surface markers panel					
	hAFMSCs (% Pos)	hAECs (% Pos)	hBMSCs (% Pos)		hAFMSCs (% Pos)		hAECs (% Pos)		hBMSCs (% Pos)	
					IFN-*γ* (−)	IFN-*γ* (+)	IFN-*γ* (−)	IFN-*γ* (+)	IFN-*γ* (−)	IFN-*γ* (+)
CD105	58.10 ± 7.15	45.27 ± 6.27	95.37 ± 4.90	HLA-ABC	99.46 ± 0.39	99.80 ± 0.17	99.53 ± 0.31	99.53 ± 0.31	97.17 ± 1.06	97.61 ± 1.47
CD90	91.67 ± 4.50	83.53 ± 12.24	91.60 ± 3.50	HLA-DR	0.59 ± 0.26	54.82 ± 1.83	0.56 ± 0.17	0.68 ± 0.29	3.37 ± 1.36	94.47 ± 8.80
CD44	86.87 ± 4.90	32.27 ± 8.05	88.70 ± 6.35	HLA-G	0.79 ± 0.51	1.16 ± 0.50	2.27 ± 1.09	11.66 ± 11.03	1.17 ± 0.43	1.29 ± 0.75
CD34	0.06 ± 0.07	0.14 ± 0.18	0.13 ± 0.07	HLA-E	1.26 ± 0.14	62.78 ± 19.07	1.93 ± 0.79	97.63 ± 1.50	2.10 ± 0.71	33.83 ± 10.18
CD45	0.09 ± 0.09	0.16 ± 0.25	0.22 ± 0.15	PD-L1	88.51 ± 4.82	99.47 ± 0.59	98.93 ± 0.91	99.70 ± 0.26	9.85 ± 5.61	98.80 ± 0.62
SSEA4	8.10 ± 4.84	52.47 ± 11.82	0.04 ± 0.04	PD-L2	34.26 ± 14.01	64.72 ± 14.63	27.63 ± 15.48	59.10 ± 27.55	10.37 ± 4.54	23.57 ± 14.69
SSEA3	14.73 ± 8.00	46.87 ± 4.30	1.38 ± 0.82	TLR5	1.00 ± 0.32	3.48 ± 2.27	0.43 ± 0.22	0.67 ± 0.20	0.63 ± 0.04	1.52 ± 1.57
				TLR6	0.55 ± 0.25	0.64 ± 0.07	0.55 ± 0.16	0.56 ± 0.16	0.58 ± 0.13	0.70 ± 0.16
				FasL	0.48 ± 0.22	0.79 ± 0.13	0.75 ± 0.55	0.62 ± 0.14	0.58 ± 0.19	0.82 ± 0.11
				Fas	58.98 ± 4.33	97.04 ± 0.89	21.10 ± 7.93	40.43 ± 7.98	11.15 ± 4.38	22.78 ± 7.30
