# Supplementary material for: Comparative Investigation of Human Amniotic Epithelial Cells and Mesenchymal Stem Cells for Application in Bone Tissue Engineering
Source: Stem Cells Int. 2015 Mar 5;2015:565732. doi: 10.1155/2015/565732 (PMC4365333; doi:10.1155/2015/565732)
Supplement: Supplementary file 1 — Microarray analysis of genes differentially expressed in hAECs, hBMSCs and hAFMSCs before and after osteogenic induction revealed 65 genes involved in ossification. [file 565732.f1.pdf]

Supplemental material I: microarray analysis method

Total RNAs was quantified by the NanoDrop ND-2000 (Thermo Scientific) and the RNAs integrity was assessed using Agilent Bioanalyzer 2100 (Agilent Technologies). The sample labeling, microarray hybridization and washing were performed based on the manufacturer's standard protocols. Briefly, 1 mg of the total RNAs were transcribed to double strand cDNAs and then synthesized cRNAs. Next, 2nd cycle cDNAs were synthesized from cRNAs. Followed fragmentation and biotin labeling, the 2nd cycle cDNAs were hybridized onto the microarray. After washing and staining, the arrays were scanned by the Affymetrix Scanner 3000(Affymetrix).

Raw expression data were normalized and subsequently analyzed with GeneChip Command Console (version 4.0, Affymetrix) software and Expression Console (version1.3.1, Affymetrix) software. Comparative genes expression analyses were performed with the Genesrping software (version 12.5; Agilent Technologies). Differentially expressed genes of hAECs, hBMSCs and hAFSCs before and after osteogenic induction were then identified through fold change. The threshold set for up- and down-regulated genes in per pair-wise comparison was a fold change>= 1.5. Functional classification of the selected genes involved in osteogenesis and ossification was conducted using the Gene Ontology Annotation database. Both hierarchical cluster analysis and protein interaction network analysis were performed on the up-regulated genes of hAECs, hBMSCs and hAFSCs involved in osteogenesis and ossification using the MultiExperiment Viewer software (Version: v4.9; Dana-Farber Cancer Institute) and the search tool STRING (http://string-db.org). For protein interaction network analysis, a gene of interest was classified as a hub if it had more than 5 interactions with other genes. To increase the result validity, the analysis was restricted to include only experimentally determined protein interactions, database annotations and textmining.

Supplemental material II: Primer sequences used for real-time PCR analysis

Gene	Primer Sequence		Accession Number
GAPDH	Forward	GCTCTCCAGAACATCATCC	NM_002046.3
	Reverse	TGC TTCACCACCTTCTTG	
COL I	Forward	CAGCCGCTTCACCTACAGC	NM_000088.3
	Reverse	TTTTGTATTCAATCACTGTCTTGCC	
ALP	Forward	ACTCCCACTTCATCTGGAACC	J04948.1
	Reverse	CCTGTTCAGCTCGTACTGCAT	
OPN	Forward	CAGAATGCTGTGTCCTCTGAA	NM_001040058
	Reverse	GTCAATGGAGTCCTGGCTGT	
Osterix	Forward	TGAGGAGGAAGTTCACTATGG	BC101549.1
	Reverse	TTCTTTGTGCCTGCTTTGC	
Runx2	Forward	GTCTCACTGCCTCTCACTTG	BC108920.1
	Reverse	CAC ACATCTCCTCCCTTCTG	
BMP2	Forward	GGGCATCCTCTCCACAAA	NM_001200.2
	Reverse	GTCATTCCACCCCACGTC	
BMP4	Forward	CCACGAAGAACATCTGGAGAAC	BC020546.2
	Reverse	ATACGGTGGAAGCCCCTTT	
BMP6	Forward	TGCAGGAAGCATGAGCTG	NM_001718.4
	Reverse	GTGCGTTGAGTGGGAAGG	
FOXO1	Forward	CGCAGATCTACGAGTGGATGGT	NM_002015.3
	Reverse	GCTCGGCTTCGGCTCTTA	
FOXC1	Forward	CACACCAGCGAACAGAAT	NM_001453.2
	Reverse	GCAAGGAAGAAGGCAAGA	
FOXC2	Forward	GCCTAAGGACCTGGTGAAGC	NM_005251.2
	Reverse	TTGACGAAGCACTCGTTGAG	
